# All-optical coherent population trapping with defect spin ensembles in silicon carbide

**DOI:** 10.1038/srep10931

**Published:** 2015-06-05

**Authors:** Olger V. Zwier, Danny O’Shea, Alexander R. Onur, Caspar H. van der Wal

**Affiliations:** 1 Zernike Institute for Advanced Materials, University of Groningen, NL-9747AG Groningen, The Netherlands

## Abstract

Divacancy defects in silicon carbide have long-lived electronic spin states and sharp optical transitions. Because of the various polytypes of SiC, hundreds of unique divacancies exist, many with spin properties comparable to the nitrogen-vacancy center in diamond. If ensembles of such spins can be all-optically manipulated, they make compelling candidate systems for quantum-enhanced memory, communication, and sensing applications. We report here direct all-optical addressing of basal plane-oriented divacancy spins in 4H-SiC. By means of magneto-spectroscopy, we fully identify the spin triplet structure of both the ground and the excited state, and use this for tuning of transition dipole moments between particular spin levels. We also identify a role for relaxation via intersystem crossing. Building on these results, we demonstrate coherent population trapping -a key effect for quantum state transfer between spins and photons- for divacancy sub-ensembles along particular crystal axes. These results, combined with the flexibility of SiC polytypes and device processing, put SiC at the forefront of quantum information science in the solid state.

Strong interaction between a long-lived spin state and an optical field is a powerful resource for field-sensing^1^,^2^,^3^,^4^ and quantum information^5^,^6^ applications. Coherent population trapping (CPT) of spins^7^,^8^,^9^ is here fundamental to all-optical control. When the interaction with a spin is weak, addressing an ensemble of identical spins can give a collectively-enhanced^10^,^11^,^12^, strong interaction. For work with solids, favorable spin properties were identified for the nitrogen-vacancy defect in diamond^8^,^13^,^14^,^15^,^16^ and divacancies in SiC^17^,^18^,^19^,^20^,^21^,^22^,^23^,^24^. However, for such defects inhomogeneities often impede resonant optical addressing of ensembles. Besides inhomogeneity for the optical transition frequencies, defects show a distribution of orientations in the crystal. This prohibits homogenous interaction with fields since the orientation sets the direction of the electric dipole moment and crystal field for spin. The diatomic layering of SiC offers here an advantage over diamond in that it partly removes degeneracies for defects in different orientations^19^,^20^. Here we demonstrate all-optical addressing and CPT for spin states of SiC divacancies, selectively on ensembles in particular crystal directions. CPT was realized after using two-laser control techniques for identifying the spin *S* = 1 structure of the ground and optically excited states, and a role for relaxation pathways via intersystem crossing^25^. Our results show that defect spin ensembles in SiC are promising systems for advancing the aforementioned applications, including the technically robust spin-ensemble approaches^10^ to photon-mediated quantum networks with spins in solid state^5^. With the well-developed semiconductor processing for SiC^26^,^27^ and the near-telecom value for the divacancy optical transition wavelength, research on integrated quantum device structures with existing technologies is within reach.

The divacancies (missing neighboring carbon and silicon atoms, here denoted as *V*_*SiC*_) occur naturally in our high-purity, semi-insulating wafer, which was obtained commercially (Methods). Figure 1a presents the lattice and possible *V*_*SiC*_ orientations in the 4H polytype we work with. Basal-plane *V*_*SiC*_ occur in six different directions, as indicated in the grid at the bottom. These have equivalent crystal environments and (for zero magnetic field) identical optical transition frequencies. The *V*_*SiC*_ along the *c*-axis have different transition frequencies^19^,^20^, and are thus an obvious choice for addressing unidirectional ensembles. Our experiments focus nevertheless on basal *V*_*SiC*_ since this allows for exploiting symmetries between *V*_*SiC*_ in particular directions. We study how an external magnetic field can either define or break these symmetries, which is of interest for field-sensing applications. Specifically, we selectively address sub-ensembles of basal *V*_*SiC*_ in particular directions by applying a weak magnetic field in the basal plane, in Fig. 1a parallel to the two orange arrows (defining sub-ensemble *P*, Parallel), and hence at 60 degrees to the four green arrows (sub-ensemble *R*, Rotated). Small misalignment angles are labeled *θ* and *φ* . Due to the anisotropy of the spin *S* = 1 Hamiltonian for *V*_*SiC*_ the *P* and *R* sub-ensembles respond differently to the applied field. However, for zero misalignment, the symmetry within these sub-ensembles gives a homogenous response to the applied magnetic field and laser driving for both *P* and *R*.

For the electronic ground (g) and excited (e) state of *V*_*SiC*_, the spin Hamiltonian has the form^18^,^20^


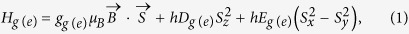


where *g*_*g*(*e*)_ is the g-factor, *μ*_*B*_ is the Bohr magneton, 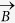 is the applied magnetic field, 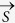 is the unitless spin *S* = 1 operator, and *h* is Planck’s constant. *D*_*g*(*e*)_ and *E*_*g*(*e*)_ are the crystal-field splitting parameters in Hz, from spin-spin interaction and crystal anisotropy, respectively. The *z*-axis points along the divacancy axis from the missing Si-atom to missing C-atom position.

Figure 1b illustrates the energy eigenstates 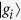 and 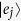 (with *i*,*j* = *l*[*ower*], *m*[*iddle*], *u*[*upper*]) of equation (1), which are superpositions of pure spin *S*_*z*_ states. The nine double-headed arrows indicate the possible direct optical excitation and decay pathways, and we will use blue, black and red colouring for transitions that couple to 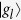, 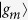
and 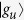 respectively. Gray arrows indicate alternative non-radiative decay paths from levels 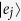 to 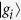 via a singlet state 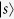
25. This process, known as intersystem crossing (ISC), also occurs for nitrogen-vacancy (NV^−^) centers in diamond^28^ where it can yield high-fidelity spin initialization due to preferred relaxation into 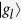. Finally, divacancies can bleach under optical excitation (not depicted in Fig. 1b). Here the divacancy alters its charge state and becomes off-resonant with the driving lasers (also found for NV^−^ centers^29^). Our measurements show laser-frequency selective bleaching that can last for hours, and that it can be rapidly reversed with a 685-nm repumping laser (Supplementary Information). Notably, such selective bleaching can be applied for removing inhomogeneity for the optical transitions^30^ (but we do not apply this in our present study).

For initial characterization of our material we studied the photoluminescence spectrum from above-bandgap laser illumination (380 nm) at 12 K (Fig. 1c). Several sharp zero-phonon lines (ZPL) from divacancy defects in different crystal environments come from the direct optical decay between levels as in Fig. 1b. Each of these lines is accompanied by a broad phonon sideband, stretching to lower energies. The blue-shaded ZPL near 1.15 eV (known as PL4^19^) belongs to the basal *V*_*SiC*_ we focus on. Next, we resonantly address an ensemble of these divacancies with excitation lasers tuned to this ZPL, and collect light emitted in the phonon sideband between 1.145 and 1.120 eV (purple-shaded part Fig. 1c). When scanning a laser across this ZPL the phonon-sideband emission is proportional to the excitation into levels 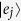, and the resulting spectrum has a resolution set by the laser accuracy (1 MHz): a technique known as photoluminescence excitation (PLE). The inset in Fig. 1c shows such a PLE spectrum from scanning a single laser across the PL4 line at 16 K, revealing a ZPL that has an inhomogeneous width of 30 GHz, which smears out the spectral fingerprint of particular 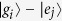 transitions. We attribute this inhomogeneity to strain in the sample^21^.

For investigating the spin-related fine structure within the ZPL, we use a two-laser spectroscopy technique^13^,^31^ that gives spectral features that are governed by the homogeneous optical linewidth. It reveals PLE signals from a sub-ensemble (here not in the sense of *P*, *R*, but with respect to inhomogeneity for the transition) of *V*_*SiC*_ with homogeneous transition frequencies. We exploit that our system only gives high PLE signal when one laser is simultaneously resonant with transitions from two different 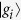 levels, while the other laser is resonant with a transition from the third 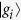 level (Methods). At these fields and laser detunings optical pumping into one of the long-lived levels 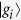 is prevented, such that the PLE from this particular sub-ensemble does not darken. Magneto-spectroscopy results of such two-laser studies are presented in Fig. 2a, obtained with one laser fixed and the other scanning near-central on the inhomogeneously broadened ZPL, and the sample at 4.2 K. The result shows several bright PLE lines, which identify points where transition energies from two different levels 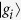 are identical to within their homogeneous linewidths. This occurs in particular for the levels 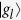 and 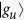, as illustrated in Fig. 2b–d. Figure 2b shows the calculated evolution of the 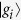 and 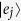 levels with magnetic field (using parameters derived from our measurements, see below). Figure 2c shows the corresponding optical transition frequencies, where the width of the traces represents the transition linewidth (Supplementary Information). For many magnetic field values, transitions that couple to 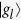 (blue) and 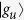 (red) are nearly equal. This yields optical excitation schemes as in Fig. 2d. By varying the field alignment, the overlap can be optimized, making the PLE lines more distinct.

The curved PLE lines in Fig. 2a allow for a detailed analysis of the parameters *D*_*g*(*e*)_ and *E*_*g*(*e*)_ of equation (1). This also yields detailed insight into the spin overlap 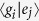 which governs the strength of a 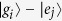 optical transition, and thereby the amplitude of the PLE signals (Franck-Condon principle with respect to spin^32^). We fit the results of Fig. 2a with a model (Supplementary Information) that combines rate equations for transitions with solving equation (1). The results are presented in Fig. 2e, with green and orange shading representing PLE from the sub-ensembles in *P* and *R* orientations.

The fit closely resembles the data in Fig. 2a in nearly all features. For example, the increase in PLE background around 38 mT results from a single laser pumping from all three ground states at that particular field for sub-ensemble *P* (the point where transitions of three colours cross in the center of Fig. 2c). With *D*_*g*_ = 1.334 GHz and *E*_*g*_ = 18.7 MHz from literature^19^ (consistent with our measurements), the fitting yields excited-state parameters *D*_*e*_ = 0.95 ± 0.02 GHz and *E*_*e*_ = 0.48 ± 0.01 GHz, and a rate *G*_0_ = 20 ± 5 MHz for the radiative contribution to the homogeneous linewidths. Getting detailed agreement between the modeling and the data required inclusion of intersystem crossing rates between 1 and 14 MHz, with a dependence on magnetic field (Supplementary Information). The analysis for Fig. 2e also identifies for each PLE line whether the underlying pumping scheme is of the Π or Λ type (defined in Fig. 2d).

We next show that our two-laser addressing of systems with a three-level ground state is suited for coherent control of the spin states. Of particular relevance is coherent population trapping (CPT), a key effect in quantum-optical control of spins^7^. Here, two-laser driving of two states 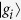 to a common state 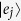 shows —on exact two-photon resonance— destructive quantum interference in the dynamics to the excited state, which results in coherent control of the ground state. Specifically, for the Λ driving scheme as in Fig. 2d, the system would (in the case of ideal spin coherence) get trapped in the state


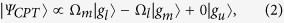


where Ω_*l*_ and Ω_*m*_ are the Rabi frequencies for the driven transitions from 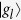 (blue-red arrow) and 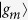 (black arrow), respectively. Notably, this example uses again that the doubly-resonant laser avoids population trapping in 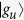. This can be directly applied in schemes where the blue-red arrow in Fig. 2d is a control field and the black arrow a signal field.

For studying the occurrence of CPT, we focus on the PLE lines labeled Λ_1_ in Fig. 2e. Figure 3 presents PLE spectra taken at the locations marked I through V in Fig. 2a, with both data for orientations *P* (orange markers) and *R* (green markers). Panel I shows how a central dip appears in the PLE line for sub-ensemble *P* as the laser power is increased. This is the spectral signature of CPT, where trapping in a ground-state superposition causes a quenching of the optical excitation. For our measurements the amplitude of the dip in the PLE signal is suppressed due to our experimental geometry, which gives decaying intensities for the lasers fields while they propagate in the sample (Supplementary Information).

For the observed CPT dips we find good agreement with calculations (solid lines in Fig. 3, Supplementary Information). This yields a ground-state dephasing time of 42 ± 8 ns, which is about 30 times shorter than previously reported from electron-spin-resonance studies on a comparable sample^19^. Such a discrepancy is likely due to extra dephasing arising from the permanent strong laser driving, which causes a fluctuating Stark effect^21^ from charges that move in and out of localized traps, as also reported for NV^−^ centers^9^. This can be avoided by using pulsed laser control or reduced powers, which is inherent to the envisioned quantum applications of *V*_*SiC*_^8^,^9^. Additionally, the observed CPT feature appears partly broadened by the 1 MHz standard deviation in determining the laser frequencies.

In panels II through V, the evolution of the CPT dip with magnetic field is shown for both the *P* and *R* sub-ensembles for constant laser powers of 3 mW. The CPT dip clearly splits at higher magnetic fields. This is caused by small misalignment angles of *θ* = 0.8 ± 0.2^°^ and *φ* = 1.8 ± 0.2^°^ that break the symmetry between the two (four) different defect orientations within the *P* (*R*) sub-ensemble. We confirmed this by tuning the misalignment angles (Supplementary Information). Due to their different symmetries, splittings in *P* are particularly sensitive to variations in *θ*, while splittings in *R* respond predominantly to variations in *φ*. For optimized sample geometries, and with the reported spin coherence times^19^,^23^, the ultimate accuracy for probing the CPT-dip frequencies should be at the kHz level^7^. Sensitivity to field alignment varies between 15 and 25 MHz per degree for different CPT splittings at 70 mT (Supplementary Information), which gradually decreases for lower fields. Here the CPT sensitivity to field magnitude is ~30 MHz/mT.

Our results thus identify the potential for unidirectional divacancy ensembles for CPT-based field sensing and quantum information applications. Compared to NV^−^ centers, currently the most investigated colour center for quantum applications^15^,^16^, it shows comparable performance where it depends on spin coherence or optical transitions, while being better compatible with existing technologies for device fabrication, and having better prospects for integration into existing telecommunication networks. Taken together with recent investigations into other defects with similar spin properties, e.g. silicon vacancies in diamond^33^ and in SiC^24^, our work illustrates the emergence of a larger set of colour centers that have potential for spintronic applications.

## Methods

### Sample fabrication

The sample was cut from a 365-*μ*m thick wafer, purchased at CREE Electronics, product HPSI W4TRD0R-0200 (http://www.cree.com/). The sample geometry was optimized for PLE detection (Supplementary Information). To cleave it into its precise final shape, a diamond-tipped stylus made a shallow groove in the wafer, after which it was broken along the groove by manually applying force with tweezers. A 100-nm gold mirror coating was evaporated onto the front and back of the sample, with a 1-nm layer of titanium for adhesion. We used a hard mask to keep a small region free for the lasers to couple into the sample.

### Experimental setup

The sample was placed in a liquid-helium flow cryostat, where the sample temperature was kept stable to within 0.01 K with a PID controller managing the helium flow and a sample heater. Windows on the four sides of the square cryostat allowed for optical access to the sample. Two tunable, continuous-wave diode lasers provided excitation light near the ZPL wavelength 1078.6 nm. We present here results taken with equal powers for the two lasers. The lasers had a linewidth below 1 MHz, and were stable to within 30 MHz over 10 minutes. This was characterized by having the two lasers interfere on a photodetector, and monitoring the shape of the beat envelope, and time-evolution of the beat frequency, caused by a minor detuning of the lasers. The two lasers beams entered the sample with identical linear polarization, and we did not observe a dependence on changing this polarization, which is expected given the low defect symmetry. Laser powers were varied between 30 *μ*W and 3 mW with neutral density filters. To counter bleaching, we added a 685 nm diode laser which was permanently on.

The frequencies of the two tunable lasers were monitored using a wavelength meter, containing several staged interferometers, which could be applied for determining frequencies with a standard deviation of 1 MHz. The sample was mounted facing a window where excitation light entered, while the detected PL(E) emission left the sample at 90^°^ from the excitation lasers. This light was collected by a 1.2-cm focal length lens mounted inside the cryostat. Outside, it was sent through additional long-pass filters with a 1082-nm cut-off to remove remaining light from the excitation lasers, and focussed into an 800-*μ*m core multimode fiber. This was connected to a spectrometer to measure PL, or to an InGaAs single photon counter (range 900 to 1150 nm) for PLE. The magnetic field was generated by a superconducting magnet, and was fixed along the direction of PL collection, in the plane of the sample.

### Measurement techniques

**Figure 2:**
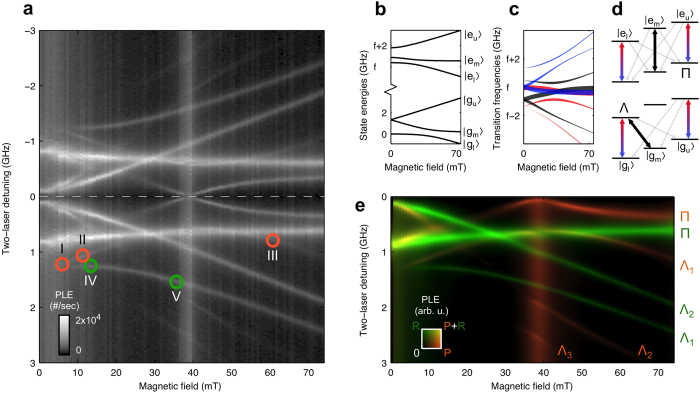
Two-laser magneto-spectroscopy of spin-related fine structure in the PL4 transition. (**a**) PLE emission of the divacancies as a function of the frequency difference between the applied lasers, and applied magnetic field. The symmetry around 0 GHz detuning is inherent to the method (see Methods). Labels at coloured markers refer to Fig. 3. (b) Calculated energy levels of the ground and excited state spin *S* = 1 systems, separated by transition frequency *f*, as a function of magnetic field (here for defect orientations *P*). The traces reflect the competition between the magnetic and crystal field terms in equation (1). (**c**) Based on panel (**b**) the frequencies of the nine transitions of Fig. 1b as a function of magnetic field (for orientations *P*). The colour-coding of Fig. 1b is used again to specify the involved 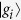. The width of traces represents the transition linewidth. To get bright PLE, optical pumping into one of the three long-lived ground-state levels 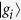 has to be avoided by having lasers resonant with a red, black and blue transition. Where two transitions of different colour overlap (for example, the blue and red transitions near the central frequency *f*) this can be realized with two lasers. (**d**) Examples of two-laser pumping schemes where one laser (blue-red arrow) is resonant with two transitions (blue and red from panel **c**) while the other laser (black arrow) is resonant with one transition. Gray arrows are decay paths. Schemes where the lasers (do not) couple two different levels 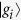 to the same level 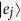 are termed Λ (Π). (**e**) Calculated PLE levels from fitting our theoretical modeling to the data in panel (**a**). Green and orange colour indicate emission from the *P* and *R* sub-ensembles. Lines are labeled according to their Λ or Π pumping scheme.

**Figure 3:**
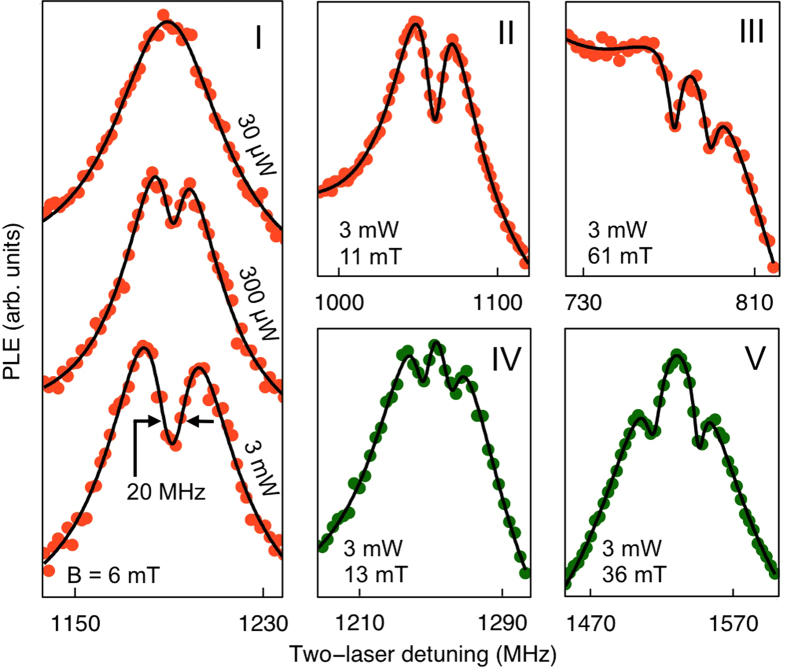
Spectral signatures of coherent population trapping. Panels I through V show dips from coherent population trapping (CPT) in PLE spectra taken at locations I-V in Fig. 2a (applied laser powers and magnetic fields as indicated). Panel I shows the emergence of a CPT dip with increasing laser power, in the two-laser PLE lines (offset for clarity) for the Λ_1_ line of defect orientations *P* (orange). Panels II through V present CPT dips in the Λ_1_-lines for both *P* and *R* orientations at increasing magnetic field, showing a gradual splitting of the CPT feature. This reflects small misalignments with magnetic field *θ* = 0.8^°^ and *φ* = 1.8^°^ (as defined in Fig. 1a). Solid lines are fits for a theoretical model of CPT.

For the two-laser spectroscopy data of Fig. 2, one laser was fixed central on the ZPL. The second laser was used for frequency scanning around the frequency of the fixed laser at a rate of 10 MHz/sec. For an ensemble with inhomogeneous transition frequencies, the roles of the two lasers must nevertheless be equivalent, and this underlies the symmetry around 0 GHz detuning in Fig. 2a. Frequencies of both lasers were recorded every few milliseconds, to allow for accurate frequency binning of photons counts, and quick automated correction for frequency mode hops of the lasers. The accumulated number of counts from the photon counter were read out from a time-to-digital converter once per second. This, together with the limited scan linearity, set the resolution for these scans to 10 MHz. This was then repeated for different magnetic fields. At the start of each scan, the PLE from the fixed laser was measured, and was subtracted from the data. For the traces in Fig. 3, the scanning laser was repeatedly scanned around the frequency of the fixed laser over a range of a few hundred MHz, with 7 s scan duration. To obtain a higher frequency resolution than in the experiment for Fig. 2, the arrival time of each photon was correlated with the frequency measurement times, allowing for a minimum precision of 2 MHz, limited by the frequency measurement standard deviation of 1 MHz.

## Additional Information

**How to cite this article**: Zwier, O. V. *et al.* All-optical coherent population trapping with defect spin ensembles in silicon carbide. *Sci. Rep.* **5**, 10931; doi: 10.1038/srep10931 (2015).

## Supplementary Material

Supplementary Information

## Figures and Tables

**Figure 1:**
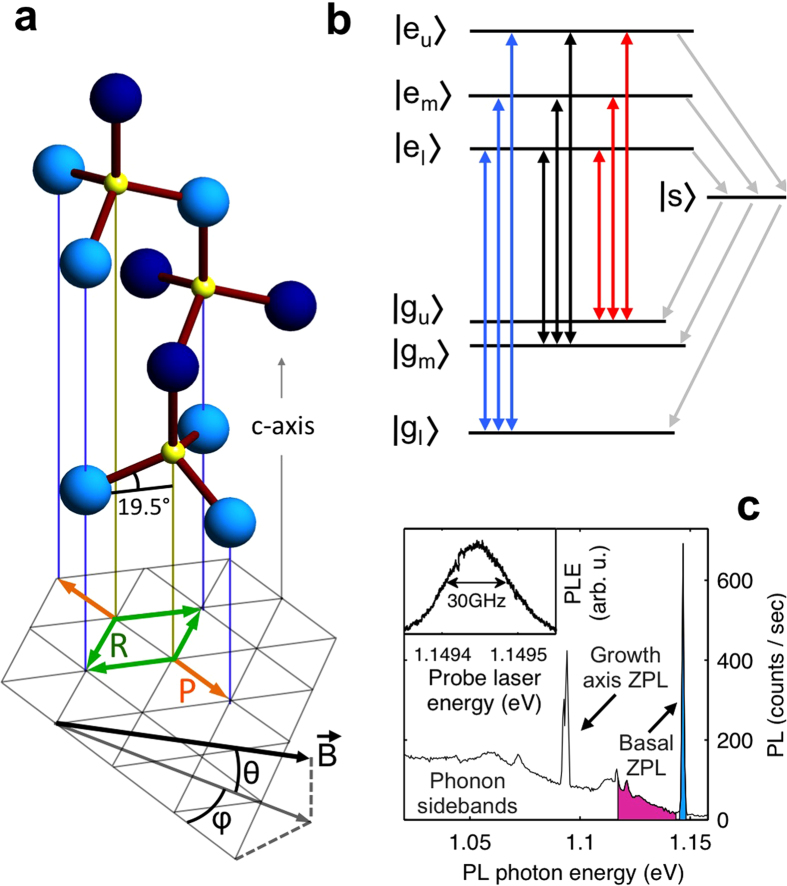
Crystal structure, energy levels, and optical signatures of divacancy defects in 4H-SiC. (**a**) In the 4H-SiC crystal structure one can recognize carbon (yellow)-centered tetrahedrons with four silicon atoms (blue) at the corners. Dark and light blue signify silicon layers that have different crystal environments. This gives different optical transition energies for *V*_*SiC*_ along the *c*-axis (vertical) and *V*_*SiC*_ in the other six directions (basal-plane *V*_*SiC*_, projections indicated on the bottom plane in orange, green, for sub-ensembles *P*, *R*, respectively). The magnetic field is applied as indicated, parallel to the orange *V*_*SiC*_ projections (with small misalignment angles *θ*, *φ*). Laser beams propagate near-parallel with the *c*-axis (Methods, Supplementary Information). (**b**) Level structure for the transitions of the zero-phonon line (ZPL) for basal *V*_*SiC*_. The ground state and excited state both have a triplet *S* = 1 spin structure (see main text for details). Vertical arrows indicate the nine possible optical transitions, where colouring labels the involved ground-state level 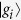. Gray lines indicate inter-system-crossing (ISC) relaxation pathways (via a singlet state 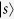). (**c**) Photoluminescence (PL) from 4H-SiC at 12 K, showing ZPL of *V*_*SiC*_ in different crystal environments and their overlapping phonon sidebands (PSB). The blue-shaded ZPL (known as PL4) belongs to the basal *V*_*SiC*_. In photoluminescence-excitation (PLE) studies lasers are resonant with PL4, while photons emitted in the PSB (pink) are used for detection. Inset: PLE spectrum of the PL4 line reveals an inhomogeneous linewidth of 30 GHz.
