# Supplementary material for: All-optical coherent population trapping with defect spin ensembles in silicon carbide
Source: Sci Rep. 2015 Jun 5;5:10931. doi: 10.1038/srep10931 (PMC4456942; doi:10.1038/srep10931)
Supplement: Supplementary Information [file srep10931-s1.pdf]

Supplementary Information for

All-optical coherent population trapping with defect spin ensembles in silicon carbide

Olger V. Zwier,[1] Danny O'Shea,[1] Alexander R. Onur,[1] and Caspar H. van der Wal[1]

[1] *Zernike Institute for Advanced Materials,*
University of Groningen, NL-9747AG Groningen, The Netherlands

(Dated: Version of April 8, 2015)

Contents

References 3

1. Sample design 4

2. Rate model of two-laser spectroscopy 5

3. Modeling of Coherent Population Trapping 11

4. Angular dependence of CPT 12

5. Bleaching and charge state dynamics 13

1. Friedrichs, P., Kimoto, T., Ley, L. & Pensl, G. (Eds.) *Silicon Carbide* Volume 1 & 2 (Wiley, 2010).
2. Fox, M. *Optical Properties of Solids* (Oxford University Press, 2nd edition, 2010).
3. Koehl, W. F. *et al.* Room temperature coherent control of defect spin qubits in silicon carbide. *Nature* **479**, 84 (2011).
4. Falk, A. L. *et al.* Polytype control of spin qubits in silicon carbide. *Nature Communications* **4**, 1819 (2013).
5. Goldman, M. L. *et al.* Phonon-Induced Population Dynamics and Intersystem Crossing in Nitrogen-Vacancy Centers. Preprint at http://arXiv.org/abs/1406.4065 (2014).
6. Fleischhauer, M., Imamoglu, A. & Marangos J. Electromagnetically induced transparency: Optics in coherent media. *Rev. Mod. Phys.* **77**, 633 (2005).
7. Beha, K. *et al.* Optimum photoluminescence excitation and recharging cycle of single nitrogen-vacancy centers in ultrapure diamond. *Phys. Rev. Lett.* **109**, 097404 (2012).

1. SAMPLE DESIGN

We designed a sample structure that gave a high amount of PL and PLE emission, optimized PL(E) collection efficiency, while also geometrically separating the weak divacancy emission from the much stronger control laser beams (Fig. S1). Gold coatings of 100 nm thickness on the front and back of the sample act as mirrors that trap the applied laser beams in the sample. This enhances the path length of the laser in the sample, giving a higher number of divacancy defects that contribute to the emission signal. The laser beams enter the sample at the top-right corner (window of 0.3 mm by 0.2 mm without gold coating), while the sample has a 45° forward tilt with respect to the laser beam. Due to refraction at the interface this gives propagation in the sample that is near-parallel to the c-axis, with a deviation that is just large enough for trapping the light on a non-overlapping zig-zag trajectory. We measured a 10% intensity loss at each reflection. In this manner laser light propagates until it reaches the left facet of the sample (no gold coating), denoted by an x, where most of the laser light still does not leave the sample due to total internal reflection. A small fraction does exit because the interfaces are not perfectly smooth. This approach give a suppression of 10^6 in laser light reaching our detectors. Conversely, divacancy emission is in random directions and for a large part on trajectories that do leave the sample when reaching the left facet. This includes trajectories that first get reflected inside the sample, and this is enhanced by total internal reflection at the trapezoidal shape on the right of the sample. This gives PL and PLE emission that mainly exits the sample on the left, in a cone of directions, facilitating efficient detection. We apply additional spectral filtering before our detectors for reaching an overall suppression of over 10^{10} for light from the excitation lasers.

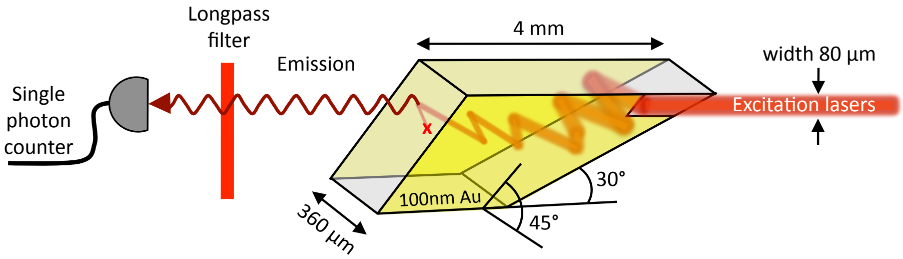

FIG. S1: **Multipass SiC sample.** Schematic of the sample geometry used in our experiment. Lasers are incident on the sample from the right, entering through a window of 0.3 mm by 0.2 mm. Light propagates as shown, hardly exiting the sample at the marker x due to total internal reflection. This results in a factor 10^6 suppression for laser light reaching PL(E) signal detectors. Light emitted by the divacancies does exit the sample, on the left. After additional spectral filtering it is collected by a single photon counter (or spectrometer). The trapezoidal shape of the sample, combined with gold coating on the front and back, further enhances this collection efficiency.

2. RATE MODEL OF TWO-LASER SPECTROSCOPY

We model the spectral positions and amplitudes of PLE signals for data as in Fig. 2a in an approach that combines rate equations for the transitions between the levels $|g_i\rangle$ and $|e_j\rangle$ with solving the spin Hamiltonian (equation (1)) for the ground and excited state. After fitting this yields results as in Fig. 2e. The rate equations are the set of coupled equations for the processes and parameters that are illustrated in Fig. S2, and this system's steady-state solution determines the PLE signals from our experiments with continuous excitation. We use an approach that only describes populations on the levels (and no coherences between the levels) in order to keep the total number of fitting parameters low. We repeated our experiments with variation of laser intensities over two orders of magnitude, and obtain nominally the same values for the fitting parameters (besides the trivial increase in PLE signal). This justifies that we can neglect coherences in our modeling.

The parameters for the rate equations, and the parameters D_e and E_e of the excited-state spin Hamiltonian, are obtained from fitting the model to data sets as in Fig. 2a. We find parameters D_g and E_g for the ground-state spin Hamiltonian that are consistent with literature, and we therefore use these as fixed values. Similarly, the g-factor is set to 2 for both ground and excited state (deriving the g-factors from our experimental data gives

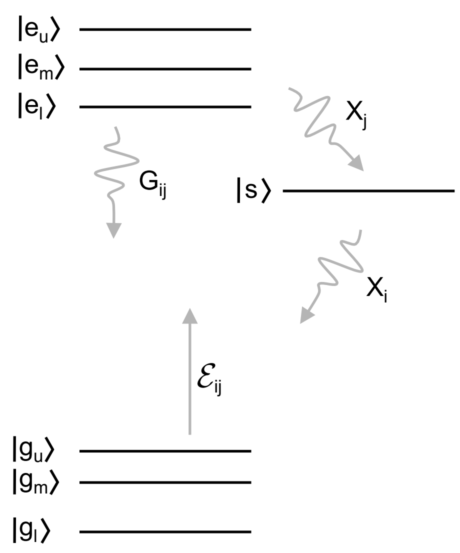

FIG. S2: **Schematic of energy levels and processes of the rate-equation model.** $\mathcal{E}_{ij}$ is the rate of laser excitation from $|g_i\rangle$ to $|e_j\rangle$, G_{ij} is the radiative decay rate from $|e_j\rangle$ to $|g_i\rangle$, X_j^e is the intersystem-crossing (ISC) decay rate from $|e_j\rangle$ to $|s\rangle$, and X_i^g is the ISC decay rate from $|s\rangle$ to $|g_i\rangle$.

agreement with published values to within the experimental error[1]). For a given magnetic field, the energy splittings between the three levels $|g_i\rangle$ and $|e_j\rangle$, and the associated spin states, are obtained by calculating eigenvalues and eigenvectors of equation (1) of the main text. The singlet state $|s\rangle$ is fixed. We calculate in parallel PLE signals from the P and R sub-ensembles, but for displaying the result in Fig. 2e we use orange and green coloring for the respective signal contributions. For the small offset angles θ and φ we had in our experiments (Fig. 1a) the differences in eigenvalues for different divacancy orientations within the sub-ensembles P and R are negligible for the present analysis.

We start with describing the influence of the spin states on the radiative decay from population in a level $|e_j\rangle$ to a level $|g_i\rangle$. We first analyze this for the case of zero-phonon-line (ZPL) transitions (below we will further discuss the role of radiative decay via phonon-sideband emission), which occurs at a rate G_{ij}^z and is given by

$$G_{ij}^z = C_1\, d_{ij}^2, \tag{1}$$

where C_1 is a constant and d_{ij} is the transition dipole matrix element. We can write this

out as

$$G_{ij}^z = C_1 |\langle \psi_i^g | \hat{d} | \psi_j^e \rangle|^2, \tag{2}$$

where $\hat{d}$ is the electric dipole operator, and the states $|\psi_j^e\rangle$ and $|\psi_i^g\rangle$ are the initial and final state for this transition, respectively. We will use the approximation that $|\psi_i\rangle$ can be treated as a product state of the spin state $|g_i\rangle$ and the orbital state $|\chi_i^g\rangle$ of the ground state: $|\psi_i^g\rangle = |\chi_i^g\rangle |g_i\rangle$. Similarly, we will use $|\psi_j^e\rangle = |\chi_j^e\rangle |e_j\rangle$ where $|\chi_j^e\rangle$ is the orbital state for the excited state. This approximation is valid for the case of very low spin-orbit interaction, and applies for our system given the low atomic masses of SiC. We thus also assume that the orbital state $|\chi_i^g\rangle$ is identical for the three levels $|g_i\rangle$, and that $|\chi_j^e\rangle$ is identical for the three levels $|e_j\rangle$. Further, we assume that at the magnetic fields of our experiments the highly confined orbital states $|\chi_i^g\rangle$ and $|\chi_j^e\rangle$ have negligible magnetic field dependence.

Since the dipole operator only works on the orbital part of a state, the expression for G_{ij}^z can now be written as

$$G_{ij}^z = C_1 |\langle \chi_i^g | \hat{d} | \chi_j^e \rangle|^2 \, |\langle g_i | e_j \rangle|^2, \tag{3}$$

and with the above assumptions $|\langle \chi_i^g | \hat{d} | \chi_j^e \rangle|$ is equal for all nine $|e_j\rangle$-$|g_i\rangle$ transitions. We can thus express G_{ij}^z as

$$G_{ij}^z = G_0^z |\langle g_i | e_j \rangle|^2. \tag{4}$$

Here G_0^z is the total decay rate via ZPL emission out of the excited state, and $|\langle g_i | e_j \rangle|^2$ is the Franck-Condon factor with respect to spin[2].

We need to account for the fact that a significant part of the radiative decay occurs via phonon-sideband emission. The ODMR work on the divacancies in SiC[3,4] shows that after phonon-sideband emission the spin state is preserved during subsequent phonon relaxation (which occurs at a fast time scale). Also, with the above assumptions, the rate of phonon-sideband emission is simply proportional to the ZPL emission for all $|e_j\rangle$-$|g_i\rangle$ transitions. We can thus simply express the total decay rate from all the possible radiative transitions as rates G_{ij} (we drop the superscript z) that obey

$$G_{ij} = G_0 |\langle g_i | e_j \rangle|^2, \tag{5}$$

and the PLE signal for a particular $|e_j\rangle$-$|g_i\rangle$ transition is proportional with G_{ij} and the steady-state population in $|e_j\rangle$. We treat G_0 as a fit parameter.

In addition to this radiative decay, we include parallel, non-radiative decay paths via the singlet state $|s\rangle$ for modeling intersystem-crossing (ISC) events. Each $|e_j\rangle$ has its own

transition rate X_j^e into $|s\rangle$, and from there the spin decays with rates X_i^g to $|g_i\rangle$. We cannot exclude that the ISC rates depend on the spin state, and hence on magnetic field. We investigated fitting both with constant (field-independent) and field-dependent ISC rates. In the latter case, we treat all six ISC rates X_i^g, X_j^e as fitting parameters which may slowly vary with magnetic field (further discussed below). During the fitting, we apply (in a self-consistent manner) that the homogeneous linewidth for the optical transitions is governed by the total decay rate Γ_j out of a level $|e_j\rangle$, which we calculate as $\Gamma_j = G_0 + X_j^e$.

The optical excitation rates $\mathcal{E}_{ij}$ are modeled in a manner similar to G_{ij}^z. Here we only need to consider ZPL transitions since the phonon-sideband excitation is negligible for laser excitation near resonance with the ZPL at the temperatures of our experiments. For excitation from a level $|g_i\rangle$ to a level $|e_j\rangle$

$$\mathcal{E}_{ij} = C_2\, d_{ij}^2\, \frac{1}{(2\pi(f - f_{ij}))^2 + \Gamma_j^2}, \tag{6}$$

where C_2 is a constant, f the laser frequency and f_{ij} the resonance frequency of the transition. The last factor in this equation describes a Lorentzian lineshape of half-width $\Gamma_j/2\pi$ for the resonance (we checked that all our experiments were in the regime where the laser intensities give negligible power broadening of the PLE lines). We implement this with a fit parameter $\mathcal{E}_0$,

$$\mathcal{E}_{ij} = \mathcal{E}_0 |\langle g_i | e_j \rangle|^2 \frac{1}{(2\pi(f - f_{ij}))^2 + \Gamma_j^2}. \tag{7}$$

The relaxation rates between ground state levels $|g_i\rangle$ are assumed to be negligible compared to the radiative decay and excitation rates (we checked that including these as parameters gave very low rates that do not improve the fits). For our fitting we subtract from the data a constant PLE background that is present from single laser driving of the inhomogeneous ensemble.

Fitting results and discussion

The fitting yields $G_0 = 20 \pm 5$ MHz, $D_e = 0.95 \pm 0.02$ GHz, and $E_e = 0.48 \pm 0.01$ GHz (and we used literature values[4] $D_g = 1.334$ GHz and $E_g = 18.7$ MHz). The value for $\mathcal{E}_0$ is always such that it gives rates $\mathcal{E}_{ij}$ well below G_0 and shows no interdependence with the other parameters. For these parameters only G_0 shows a weak dependence on the various approaches to fitting the ISC rates. Attempts to fit without accounting for ISC yields

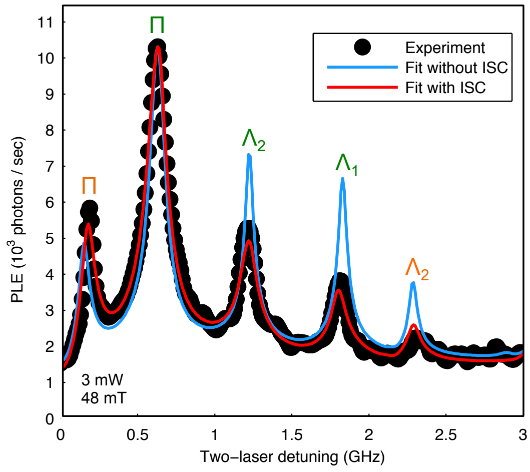

FIG. S3: **Model fits at 55 mT.** Comparison of the quality of fitting the model, with (red) and without (blue) ISC. Without ISC, the model structurally overestimates peaks attributed to Lambda schemes.

PLE lines at the correct frequencies (mainly governed by the values of the $D_{e,g}$ and $E_{e,g}$ parameters) and with the correct width (mainly governed by G_0) in Fig. 2a. However, for that case the relative amplitudes of calculated PLE lines (at a certain magnetic field value) show poor agreement with the experimental results. On this aspect we get much better agreement in an approach with magnetic-field-independent values for the six ISC rates (Fig. S3). However, we cannot exclude that the ISC rates depend on magnetic field. While the ISC processes are far from completely understood, recent theory work for diamond NV^- centers proposes a role for spin-orbit coupling, where the orbital quantization axis is linked to the defect orientation. Consequently, this work proposes that the ISC rates depend on the spin states of the levels, and thereby on magnetic field[5]. This also indicates that for non-zero magnetic field one should in fact fit with different ISC parameters for divacancies along different crystal directions. We did not explore this latter aspect since this makes the number of fitting parameters very high and we analyzed that the results would not have statistical significance for our data set. For non-zero field we thus present ISC rates that are an average over the different defect orientations.

We thus carried out a fitting approach where we used six global ISC rates that can vary as a function of field, while maintaining single parameters D_e, E_e, G_0 and $\mathcal{E}_0$ for the full data set. To suppress statistical fluctuations we effectively fit average ISC rates over magnetic field ranges of 10 mT width in Fig. 2a (over which the spins states do not strongly vary). We

then see ISC rates that show a weak variation with field, and become constant at magnetic fields over 40 mT. This can be understood by noting that this is beyond any anticrossings in the $|g_i\rangle$ and $|e_j\rangle$ levels where the spin states mix. Here, the spin states become field independent and this results indeed in stable ISC rates from the fitting.

The results for the ISC rates are presented in Table I for the applied field near $B = 0$ mT and $B > 40$ mT (the approach with field-independent ISC rates gave values between these two cases, with a least-squares error from fitting that was 20% higher). The result with these ISC rates are also presented in Fig 2e. At low fields, these ISC rates indicate relatively strong values for ISC out of levels $|e_m\rangle$ and $|e_u\rangle$, and preferential ISC decay into the level $|g_l\rangle$. This identifies how single-laser excitation into the phonon sideband or driving the ZPL with spectrally broad laser pulses (which both give parallel excitation from all spin levels, and is for example applied in ODMR studies[3,4]) results in spin polarization in the ground state.

	X_l^g	X_m^g	X_u^g	X_l^e	X_m^e	X_u^e
$B \approx 0$ mT	14±9	4±3	6±3	2±1	6±3	5±3
$B > 40$ mT	3±3	5±3	1.1±0.7	3.4±0.8	8±3	2.9±0.5

TABLE I: Fitting results for the ISC rates. All values are in MHz.

3. MODELING OF COHERENT POPULATION TRAPPING

We investigated the agreement between our CPT observations (Fig. 3) and the standard theoretical description for this phenomenon[6] (unlike the PLE modeling of the previous section, this modeling does include coherences between the levels). This also allows for extracting the ground-state spin dephasing time T_2^* for our ensemble. For making the fits for panels I-V in Fig. 3, we approximate the six-level system as an effective three-level system, where we only account for the levels that participate in a laser-driven Λ configuration (Fig. 2d). For this system we determine the steady-state solution of the density-matrix master equation, with the approach described in Ref. 6. In order to relate it to the measured data from our two-laser spectroscopy technique, we fit using a sum of master equation solutions, where the summation is over a range of inhomogeneous broadening values for the optical transition. (giving that the CPT dip is always in the middle of a PLE spectral line). In addition, we take into account the decaying power along the beam path in our multipass sample (using a decay constant that was measured), by fitting with a sum of master equation solutions with exponentially decreasing Rabi frequencies. For the measurements that show two (four) CPT dips due to small misalignment angles for the applied magnetic field, we sum the PLE from two (four) contributions. Here each contribution is calculated for one set of misalignment angles that occur within the ensemble that is addressed (representing one of the two or four orientations within ensemble P or R, respectively). In order to obtain good fits we need to account for a background signal in the PLE data, in particular for PLE lines with CPT that are on the wing of a neighboring PLE line. In the calculations we account for this with a linear-in-frequency background signal. We can thus calculate the CPT line shapes that appear in the PLE data, and the results are presented as the solid lines in Fig. 3. These results give a T_2^* value of 42±8 ns.

4. ANGULAR DEPENDENCE OF CPT

To find evidence for our interpretation that the splitting of the CPT dips in panels III-V of Fig. 3 is a result of minute magnetic field misalignment, we rotated the sample holder inside the cryostat in steps of 0.5°. This is a rotation around the vertical axis of Fig. S1, and yields a combined variation of φ and θ offsets due to the 45° forward tilt in our sample mounting, see Fig. 1a and Fig. S1. Results from these measurement are presented in Fig. S4a for sub-ensemble R, with magnetic field and laser powers as indicated. The CPT dips clearly move apart as a function of sample rotation, finally showing four separate dips at large rotations. These directly reflect the four defect orientations within the R sub-ensemble, with the symmetry broken by the sample rotation. Figure S4b presents the values of the two-laser detunings where the CPT dips occur (determined with phenomenological fitting of the data in Fig. S4a, the marker sizes indicate 68% confidence intervals). The black lines in Fig. S4b are calculated positions for the CPT dips, obtained with equation (1) for the four different orientations. A small misalignment in sample tilt was used as fit parameter, yielding a remaining misalignment of 1.1° at optimal alignment with the field (again a combination of φ and θ offsets due to the 45° tilt in sample mounting).

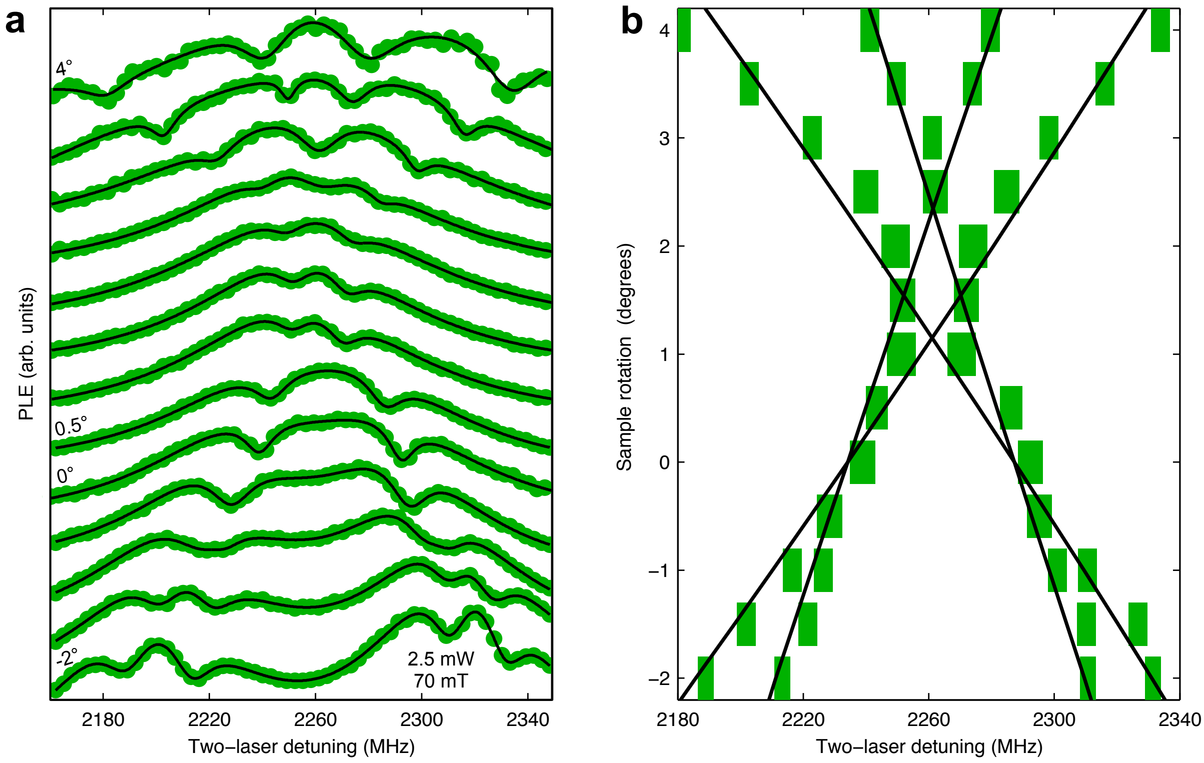

FIG. S4: **Sensitivity of CPT to magnetic field alignment.** **a**, CPT in sub-ensemble R at 70 mT and 2.5 mW power of the lasers, where the sample holder was rotated between measurements in steps of 0.5°. The splitting of the CPT dip is clearly dependent on angle, increasing when the symmetry between defect orientations is reduced. Black lines are fits made with a sum of Lorentzians. **b**, CPT detunings from the fits in Fig. S4a, where the box size indicates the confidence interval. The black lines are a fit made using the ground state Hamiltonian, where the asymmetry comes from a small tilt off the sample on its holder of 1.1°.

5. BLEACHING AND CHARGE STATE DYNAMICS

When addressing the divacancies with lasers resonant on the zero-phonon line (ZPL), the amount of PLE signal that is detected gradually decreases over time, on a timescale of minutes to hours for the laser powers we use. When keeping a single laser fixed central on the ZPL for several hours, and subsequently scanning a single laser over the entire ZPL (in a few seconds), we see a clear dip in the PLE signal (Fig. S5). This bleaching phenomenon for SiC divacancies is an effect also seen for NV^- centers in diamond, where it has been identified as a two-photon process. In case of an NV^- center, it is initially negatively charged, absorbs

two photons, and subsequently an electron is lost to the conduction band[7]. For the SiC divacancy the initial state is charge neutral, and two-photon absorption also causes the defect to make a transition to a different meta-stable charge state. In this charged state the wavelength for the ZPL optical transitions is shifted by over 50 nm. Applying an additional laser that excites these charged divacancies brings them back to their original charge state (again in analogy with observations on NV^- centers[7]). We found that applying a 300 μW repumping laser at 685 nm completely removes the bleaching dip after a few seconds, and we had such a repump laser permanently on during all the two-laser spectroscopy experiments. It is important that this laser is far-removed from the ZPL and the phonon sideband for excitation of our neutral divacancy, such that it only excites divacancies in the altered charge state. This avoids that the repump laser interferes with driving optical transitions for the neutral V_{SiC} and our two-laser spectroscopy. Notably, with selectively bleaching most of the left and right wing of the inhomogeneous linewidth of the ZPL of our neutral divacancy (the inverse of the result in Fig. S5) one can create a long-lived sub-ensemble with a strongly reduced inhomogeneity for the optical transition.

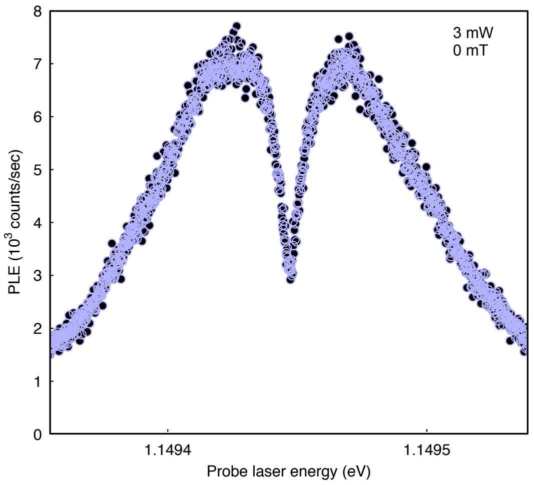

FIG. S5: **Charge-state bleaching.** The inhomogeneously broadened ZPL as in the inset of Fig. 1c, with a dip of 3.5 GHz full-width (FWHM). The dip was formed after keeping a CW laser fixed at the dip frequency, causing the PLE to steadily decrease to this value in a few hours. This effect, known as bleaching, is caused by divacancies changing their charge state. In the altered charge state the divacancies are off-resonant with the laser, and remain in this state for hours after the laser driving. This spectrum with a dip can be restored to the original peak shape (inset of Fig. 1c) within seconds by applying a 685-nm laser. This laser is exciting the charged divacancies in their phonon sideband, and this pumps them back to their neutral charge state.
